# Clinical and radiological characteristics and outcome of wake-up intracerebral hemorrhage

**DOI:** 10.1038/s41598-020-75750-x

**Published:** 2020-10-30

**Authors:** Joan Martí-Fàbregas, Raquel Delgado-Mederos, Alejandro Martínez-Domeño, Pol Camps-Renom, Daniel Guisado-Alonso, Marina Guasch-Jiménez, Paula Marrero-González, Elena Jiménez-Xarrié, Rebeca Marín, Luis Prats-Sánchez

**Affiliations:** Stroke Unit, Department of Neurology, Hospital de La Santa Creu I Sant Pau, C/Mas Casanovas, 90, 4ª planta (Secretaria Neurologia), 08041 Barcelona, Spain

**Keywords:** Stroke, Neurological disorders, Diseases of the nervous system, Medical research, Neurological disorders

## Abstract

There is little information on the characteristics of patients with wake-up intracerebral hemorrhage (WU-ICH). We aimed to evaluate frequency and relevant differences between WU-ICH and while-awake (WA) ICH patients. This is a retrospective study of a prospective database of consecutive patients with spontaneous ICH, who were classified as WU-ICH, WA-ICH or UO-ICH (unclear onset). We collected demographic, clinical and radiological data, prognostic and therapeutic variables, and outcome [(neurological deterioration, mortality, functional outcome (favorable when modified Rankin scale score 0–2)]. From a total of 466 patients, 98 (25.8%) were classified as UO-ICH according to the type of onset and therefore excluded. We studied 368 patients (mean age 73.9 ± 13.8, 51.4% men), and compared 95 (25.8%) WU-ICH with 273 (74.2%) WA-ICH. Patients from the WU-ICH group were significantly older than WA-ICH (76.9 ± 14.3 vs 72.8 ± 13.6, p = 0.01) but the vascular risk factors were similar. Compared to the WA-ICH group, patients from the WU-ICH group had a lower GCS score or a higher NIHSS score and a higher ICH score, and were less often admitted to a stroke unit or intensive care unit. There were no differences between groups in location, volume, rate of hematoma growth, frequency of intraventricular hemorrhage and outcome. One in five patients with spontaneous ICH are WU-ICH patients. Other than age, there are no relevant differences between WU and WA groups. Although WU-ICH is associated with worse prognostic markers vital and functional outcome is similar to WA-ICH patients.

## Introduction

Similar to acute myocardial infarction and sudden cardiac death, there is a circadian pattern with an increased risk of acute ischemic and hemorrhagic stroke during the early morning hours^1^. It is known^2,3^ that 8–25% of patients with ischemic stroke have symptoms first noted on awakening. Most studies^4–6^ have found no appreciable clinical or radiographic differences between wake-up and while-awake ischemic strokes. Imaging studies^4^ suggest that stroke may have occurred only shortly before awakening in a large proportion of patients and accordingly recent studies reported that selected patients with wake-up stroke benefit from intravenous thrombolysis^7^ and mechanical thrombectomy^8,9^.

In contrast to ischemic stroke, wake-up intracerebral hemorrhage (WU-ICH) has not been studied systematically. Traditionally, ICH were thought to start during waking hours and that sleep tended to facilitate ischemic but not hemorrhagic stroke^10^. The frequency of WU-ICH is uncertain and is also unknown whether there are clinical, radiological and prognostic differences between WU-ICH and while-awake ICH (WA-ICH) patients. In ICH, early comprehensive care is important since some treatments are more effective the earlier they are applied^11–13^. Therefore, detailed information about patients with WU-ICH is needed.

Our aim was to establish the frequency of WU-ICH and to compare demographic, clinical and radiological features with WA-ICH in a large series of consecutive patients admitted to a tertiary care hospital. Also, we aimed to compare neurological deterioration, mortality and functional outcome between patients with WU-ICH and those with WA-ICH. To our knowledge, only scarce information is available regarding WU-ICH patients and their comparison with WA-ICH patients^14,15^. We believe that this information may be useful for the design of future therapeutic studies.

## Methods

We conducted a retrospective study of consecutive patients of 18 years and older with spontaneous ICH included in a prospective registry. Patients were admitted from January 2015 to December 2018 to a tertiary stroke center. Patients were treated following the guidelines from the Spanish Society of Neurology^16^. The local Ethics Committee (Hospital de la Santa Creu i Sant Pau) approved the study. Due to the absence of a change in the routine management of the patients and also that the data obtained were anonymous, a signed consent from each patient or the legal representative was not deemed necessary. The same ethics committee waived the need for informed consent.

WU-ICH was defined as a patient with ICH who went to sleep normally and whose symptoms were first noted on awakening. Patients who woke up with symptoms during the night were also considered WU-ICH, but not those who woke up with symptoms after a nap. Time of onset was defined as the last-time-seen-normal. WA-ICH was defined as a patient with ICH in whom first symptoms were noted by the patient or a witness during wakefulness (morning or afternoon). We excluded patients with unclear-onset (UO-ICH), defined as those who were found unconscious or comatose during waking hours or who were unable to detail the time of onset and whose onset was not witnessed.

We collected the following variables: (1) Clinical data: demographics (age, sex); vascular risk factors; prior medication (statins, antiplatelet, oral anticoagulants); prior cognitive impairment (as assessed by anamnesis to family members or previous neuropsychological examinations); Glasgow coma scale score or National Institute of Health and Stroke Scale (NIHSS) score at admission; results of blood analyses at admission (platelet count, blood glucose, INR); blood pressure at admission; the most likely etiology; treatment data (Stroke or Intensive Care Unit admission, withdrawal of life-sustaining therapeutic measures within the first 48 h, surgical intervention, administration of prothrombin complex; lowering of blood pressure within the first 72 h; (2) Neuroimaging data: Time to admission CT (time from last-seen-well in WU patients, and from symptom-onset in WA patients); location (lobar cerebral hemisphere, deep cerebral hemisphere, infratentorial), intraventricular hemorrhage, volume (calculated with the ABC/2 formula); hematoma growth (HG, defined as an increase > 6 ml or > 33% of the hematoma volume measured at the 24 to 72 h follow-up CT compared to the admission CT; (3) ICH score at admission^17^, (4) Outcome data: Neurological deterioration (a decrease of > 1 point on the Glasgow coma scale or increase in ≥ 4 points on the NIHSS score within the first 72 h after onset); most likely cause of neurological deterioration; prior modified Rankin scale (mRS) score; mRS score at 3 months obtained after a face-to-face interview (or at discharge if the 3-month visit was unavailable); mortality within the first 3 months. A favourable outcome was defined as a score of 0, 1 or 2.

### Statistical analyses

We compared the results of categorical and quantitative variables between the WU-ICH and the WA-ICH groups. Categorical variables were expressed by number and percentages and the χ^2^ test was used to compare them between groups. Continuous variables were expressed as mean and standard deviation and comparison between groups was done using the Student’s t-test. Ordinal variables were expressed by median and interquartile range and the comparison was done using the Mann–Whitney U-test. Variables showing a trend (p < 0.1) in the univariate analysis were introduced in a multivariable logistic regression model and backward eliminated to a significance level of < 0.05. The results were considered statistically significant when the p value was < 0.05. Software for statistical analysis: SPSS v22.

### Ethics approval

The local Ethics Committee (Hospital de la Santa Creu i Sant Pau) approved the study. A signed consent from each patient or the legal representative was not deemed necessary due to the absence of a change in the routine management of the patients and also that the data obtained were anonymous.

## Results

We included 466 patients. According to the type of onset, 95 (20.3%) patients were classified as WU-ICH, 273 (58.5%) as WA-ICH and the remaining 98 (21%) as UO-ICH. We excluded UO-ICH patients from analyses. Therefore, we studied 368 patients and compared 95 (25.8%) WU-ICH with 273 (74.2%) WA-ICH. Their mean age was 73.9 ± 13.8 and 51.4% were men. Table 1 provides detailed demographic and past medical data from WU- and WA-ICH patients.

**Table 1:** Demographic variables, vascular risk factors and relevant medical history.

Variable	WU-ICH (n = 95)	WA-ICH (n = 273)	p
Age, mean (SD)	76.9 (14.3)	72.8 (13.6)	0.01
Sex, n (% men)	48 (50.5)	141 (51.6)	0.90
High blood pressure, n (%)	75 (78.9)	201 (73.6)	0.33
Diabetes mellitus, n (%)	25 (26.3)	64 (23.4)	0.58
Hypercholesterolemia, n (%)	46 (48.4)	123 (45.1)	0.63
Ischemic stroke (transient ischemic attack or infarct), n (%)	15 (15.8)	40 (14.7)	0.86
Intracerebral hemorrhage, n (%)	5 (5.3)	30 (11)	0.10
Atrial fibrillation, n (%)	22 (23.2)	51 (18.7)	0.37
Alcohol abuse, n (%)	7 (7.4)	24 (8.8)	0.83
Prior treatment with statins, n (%)	32 (33.7)	105 (38.5)	0.46
Prior treatment with antiplatelets, n (%)	34 (35.8)	80 (29.3)	0.24
Prior treatment with anticoagulants, n (%)	19 (20)	49 (17.9)	0.64
Cognitive impairment, n (%)	24 (25.3)	41 (15)	0.02
Prior mRS score, median (IQR)	2 (0–3)	0 (0–2)	0.01

Patients from the WU-ICH group were older than WA-ICH (76.9 ± 14.3 vs 72.8 ± 0.13.6 years, p = 0.010), but sex distribution and proportion of vascular risk factors were similar between groups. Frequency of cognitive impairment and prior RS score were higher in the WU-ICH group than the WA-ICH group.

**Figure 1:**
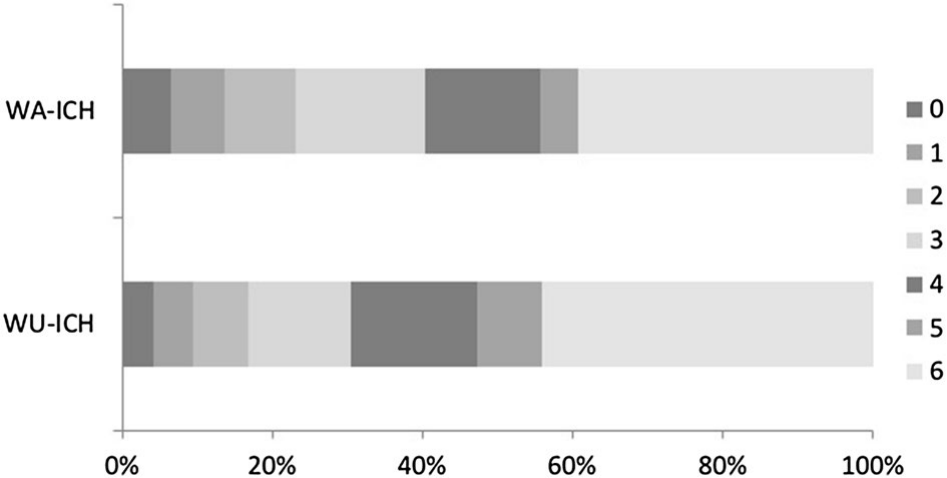
Distribution of modified Rankin scale scores in the WU-ICH and WA-ICH groups. The cell number expresses the percentage of patients with each score.

Table 2 provides clinical and blood analysis data at admission. Clinical presentation was more severe in the WU-ICH than in the WA-ICH group, as evidenced by a lower GCS score or a higher NIHSS score and a higher ICH score. The results of blood analysis and vital signs at admission was comparable between groups. Patients from the WU-ICH were less often admitted to a stroke unit or intensive care unit (55.8% vs 70.3%, p = 0.008). Other treatments and withholding of life-sustaining measures were comparable. Radiological and outcome data are detailed in Table 3. Cranial CT was obtained earlier in the WA-ICH group (median 90 min) than in the WU-ICH group (median 391.5 min), but there were no differences between groups in location, volume, frequency of HG (n = 154) and intraventricular hemorrhage. The frequency of neurological deterioration and its reasons were comparable between groups. Though patients from WU-ICH group had a worse functional outcome and higher mortality than the WA-ICH, these differences were not significant (see Fig. 1). In absolute values Table 3, the percentage of patients with mRS score 0–2 was 6.4% lower for the WU-ICH group compared to the WA-ICH group, the percentage of patients with mRS score 0–3 was 9.9% lower for the WU-ICH group compared to the WA-ICH group. In the multivariable logistic regression analysis, presenting a higher ICH score (OR = 1.25 95%CI 1.03–1.51) and the probability of being admitted in a critical care unit (OR = 0.55 95%CI 0.33–0.90) were the only variables that independently differentiate the WU-ICH group from the WA-ICH group.

**Table 2:** Clinical data, results of blood analysis at admission and therapeutic measures provided.

Variable	WU-ICH (n = 95)	WA-ICH (n = 273)	p
Glasgow coma scale score at admission, median (IQR)	14 (10–15)	15 (12–15)	0.06
NIHSS score at admission^†^, median (IQR)	15 (6–21)	11 (4–19)	0.02
Platelet count (× 109/L), mean (SD)	214.4 (119.5)	216.6 (72)	0.83
Blood glucose (mg/dl), mean (SD)	145.6 (57.4)	134 (48.3)	0.08
International Normalized Ratio (INR), mean (SD)	1.5 (1.18)	1.38 (0.93)	0.20
Systolic blood pressure at admission (mmHg), mean (SD)	168.5 (33.2)	171.1 (35.3)	0.53
Diastolic blood pressure at admission (mmHg), mean (SD)	90.6 (21.7)	88.5 (19.3)	0.39
ICH score, median (IQR)	2 (1–3)	1 (1–2)	0.02
**Etiology, n (%)**			
Hypertensive	23 (27.7)	81 (33.1)	0.08
Amyloid angiopathy	36 (43.4)	79 (32.2)	
Oral anticoagulants	17 (20.5)	38 (15.5)	
Vascular malformation	3 (3.6)	17 (6.9)	
Others/Unknown	4 (4.8)	30 (12.2)	
**Stroke Unit or Intensive Care Unit admission, n (%)**			
ICU	20 (21.1)	48 (17.6)	0.008
Stroke unit	33 (34.7)	144 (52.7)	
Conventional	42 (44.2)	81 (29.7)	
Early limitation of therapeutic effort, n (%)	20 (21.1)	43 (15.8)	0.26
Surgical intervention (any), n (%)	7 (7.4)	29 (10.6)	0.42
Vitamin K therapy in case of VKA antagonists, n (%)	12 (12.6)	32 (11.7)	0.85
Prothrombotic complex administration, n (%)	10 (10.5)	30 (11)	0.99
Intravenous blood pressure lowering agents within the first 72 h, n (%)	61 (65.7)	176 (64.9)	0.90

^†^ 268.

**Table 3:** Radiological and outcome data.

Variable	WU-ICH (n = 95)	WA-ICH (n = 273)	p
Time to admission CT, min, median (IQR)	391.5 (241.5–698.75)	90 (50–240)	0.01
**Location, n (%)**			
Deep (cerebral hemisphere)	35 (36.8)	118 (43.2)	0.53
Lobar (cerebral hemisphere)	52 (54.7)	132 (48.4)	
Infratentorial	8 (8.4)	23 (8.4)	
Intraventricular hemorrhage, n (%)	41 (43.2)	106 (38.8)	0.46
Volume at admission (ml), mean (SD)	39.1 (46.7)	30.5 (37)	0.11
Hematoma growth^†^, n (%)	12 (32.4)	37 (24)	0.30
Neurological deterioration^‡^, n (%)	35 (41.7)	113 (43.1)	0.89
**Likely cause of neurological deterioration (within 0–72 h after onset), n (%)**			
Unknown	8 (22.9)	36 (31.9)	0.24
Hematoma expansion	7 (20)	36 (31.9)	
Mass effect, intracranial hypertension	10 (28.6)	16 (14.2)	
Infectious complication	5 (14.3)	11 (9.7)	
Edema	1 (2.9)	5 (4.4)	
Hydrocephalus	2 (5.7)	2 (1.8)	
Other	0(0)	9 (8)	
mRS score at 3 months, median (IQR)	5 (3–6)	4 (3–6)	0.14
mRS score 0–2 at 3 months, n (%)	16 (16.8)	63 (23.2)	0.24
mRS score 0–3 at 3 months, n (%)	29 (30.5)	110 (40.4)	0.11
Mortality at 3 months, n (%)	42 (44.2)	107 (39.3)	0.46

^†^ n = 154.

^‡^ n = 262.

## Discussion

In a large series of consecutive patients with ICH, we compared clinical, radiological and outcome data between the WU-ICH and the WA-ICH groups. Most variables from the WU-ICH were comparable between groups. However, there were some differences between groups: First, patients from the WU-ICH group were older and had a higher frequency of prior cognitive impairment and a worse prior RS and ICH scores. Additionally, patients from the WU-ICH group had a more severe presentation, as evidenced by a higher NIHSS or lower Glasgow coma scale scores, and were less often cared in a stroke unit or an Intensive Care Unit. Because the WU-ICH group showed a higher ICH score than the WA-ICH group, we expected a worse clinical outcome in the WU-ICH group. However, the proportion of HG, neurological deterioration, unfavorable functional outcome and mortality at 3 months was similar between both groups.

Wake-up ischemic stroke occurs in about 20% of patients^18^ and multimodal neuroimaging suggests that ischemic stroke often started shortly before symptoms were recognized^4^. Does the same apply to hemorrhagic stroke? There is no clinical or radiological data that provides a reliably estimate about when bleeding started. Thus, we can trust only the report from the patient or a witness. Although we didn’t have a control group of patients with ischemic stroke, our finding that 20.3% of patients with ICH had the first identifiable symptom at the moment of awakening suggests that onset of ICH as WU-ICH is underestimated and is probably as frequent as wake-up ischemic stroke. Previous studies^10,14,19–21^. reported a frequency of WU-ICH of 13.6–19.2% but associated clinical, radiological and outcome data were not studied extensively.

The moment of onset may lead to misinterpretations. First, it may be difficult to differentiate between a patient who started bleeding during the night and wakes up with symptoms and a patient who wakes up without symptoms, but soon after waking-up suffers an ICH that renders the patient unable to ask for help. The witness would relate the symptoms as initiated at the time of awakening as the patient was well when he went to sleep. Also, the witness may be sleeping also at the onset of bleeding and thus would be unable to know that the patient is suffering a stroke. Moreover, a patient who lives alone may be unable to explain the details of stroke onset. We classified as unclear-onset up to 21% of our patients. Second, accompanying symptoms are important. Because the frequency of unpleasant symptoms, such as headache or vomiting, may be more frequent in hemorrhagic stroke than in ischemic stroke, it is conceivable that these symptoms will awake the patient at the onset of bleeding and would not go unnoticed. Conversely, because a decrease of the level of consciousness is frequent in large hemispheric or infratentorial ICH, it could be that the patient is unable to recognize the symptoms due to a decrease in alertness. We can speculate that accompanying symptoms are important only when the volume of the hematoma is high.

There is an increasing risk of ischemic and hemorrhagic stroke onset during the morning. An increase in the time of onset of ICH from 8 AM to 4 PM has been reported^22^. Likewise, it is generally assumed that sleep tends to facilitate ischemic but not hemorrhagic stroke as reported by several studies^10^ and this is attributable to changes in circadian blood pressure^1,6^. Reduction of blood pressure, heart rate, and sympathetic drive occurs during sleep. Due to the link between high blood pressure and ICH^23^, it is likely that ICH onset is associated with periods of higher BP, and therefore ICH onset is expected during waking hours. However, nocturnal hypertension occurs also in non-dippers^24^, in hypertensive patients treated with medication that do not have a prolonged effect, during transition from non–rapid eye movement (non-REM) sleep to REM sleep^25^ and in patients who suffer obstructive sleep apnea^26^. Thus, blood pressure increases during the night may explain our relatively high frequency of WU-ICH. In contrast, in our study hypertensive ICH was observed in WA-ICH more frequently than in WU-ICH, but the difference was non-significant, and blood pressure measures at admission were comparable.

Because time from onset of stroke to admission and diagnostic CT is longer in WU-ICH compared to WA-ICH, and because the risk of HG is inversely related to elapsed time^27^, it is expected that WU-ICH is associated with larger hematomas and less risk of HG as compared to WA-ICH. On the contrary, since blood pressure is a determining factor of the hematoma volume and HG^28^ the volume of the hematoma could be lower in the WU-ICH group since blood pressure is lower during sleep. In one study the mean volume was greater^14^ in the WU-ICH compared to the WA-ICH group but HG rate was not provided. Another study^15^ that compared unclear onset ICH (39% of 377 patients, combining WU-ICH and unwitnessed ICH onset) with WA-ICH patients reported larger hematoma volumes in the unclear-onset group, and a low frequency of HG in both groups. Although we found that hematoma volume was 9 mL greater in the WU-ICH group this difference was not significant and the proportion of HG and blood pressure measures at admission were not different between groups. Moreover, neurological deterioration, a surrogate marker for HG, was comparable also, as was mortality and long-term functional outcome. Since WU-ICH patients were older, with larger (although non-significant) volumes, higher neurologic severity, higher ICH score and less often received specialized care, this is an unexpected finding. In the study by Nagakene et al.^14^ age was comparable, but Glasgow coma scale was 1 point lower in the WU-ICH compared to the WA-ICH group. In agreement with our findings, RS was non-significantly higher in the WU-ICH group compared to the WA-ICH. Surprisingly and contrary to our results, they reported a very low mortality rate in the WA-ICH (4.9%), that was markedly lower than the WU-ICH mortality (21.1%, p = 0.03). A multivariate analysis also showed no differences in mortality or functional outcome in the previously mentioned study^15^ that compared unclear onset with clear onset ICH.

The patient who arrives with a known onset time and with a few hours from the onset to admission (WA-ICH) may be considered more critical or more urgent than the patient who has woken up with the symptoms (WU-ICH) and this may lead to a higher proportion of patients treated with some effective measure and to this treatment being applied with less latency from admission. Some therapeutic measures are time-sensitive^11–13,28–31^ since they are more effective the sooner they are implemented. One possible explanation for our findings is that management was the same in WU and WA patients and that effective treatment strategies despite blood-pressure lowering and hemostatic reversal in case of ICH associated to oral anticoagulants are still missing. Although admission in a stroke unit is associated with a better outcome^30^ compared to admission on a general medical ward, this may not change markedly the vital and functional outcome if the rest of therapeutic measures are provided. Our study may not have been large enough to detect this benefit. Since there was a lower proportion of favorable outcome in the WU-ICH group than in the WA-ICH group, either dichotomized as mRS 0–2 or as mRS 0–3, we cannot rule out that a larger sample of patients would have obtained statistically significant results. Our findings suggests that WU-ICH may benefit from the same therapeutic measures, although future studies should confirm this suggestion in randomized clinical trials in which WU-ICH patients are also enrolled. WU-ICH patients may not be eligible in trials with short therapeutic windows, but may be enrolled in trials with inclusion times from the onset of symptoms greater than 8 h.

Our study has some limitations. The unicentre design limits its external generalizability, although the prospective and consecutive inclusion of patients is a strength of our study. As previously discussed, the exact time of ICH onset may be misinterpreted. The exclusion of 98 patients with UO-ICH may create a selection bias and influence our estimate of the exact frequency of WU-ICH, since we do not know whether UO-ICH patients correspond to WU or WA-ICH. Estimation of HG was only possible in patients with a follow-up CT examination. However, the frequency of neurological deterioration, a surrogate marker for HG, was comparable between groups. Furthermore, although the administration of antihypertensive treatment in the acute phase was collected, the degree of control achieved with such treatment was not evaluated.

### Conclusions

Further studies are needed to assess whether WU-ICH patients and WA-ICH patients differ in etiology, pathophysiology, risk factors, and prognosis. According to our study, both groups of patients are comparable for most observable data.
